# Metabolic Bone Disease of Prematurity: Report of Four Cases

**DOI:** 10.4274/jcrpe.1282

**Published:** 2014-06-05

**Authors:** Gül Yeşiltepe Mutlu, Heves Kırmızıbekmez, Elif Özsu, İlkay Er, Şükrü Hatun

**Affiliations:** 1 Zeynep Kamil Gynecologic and Pediatric Training and Research Hospital, Department of Pediatric Endocrinology, İstanbul, Turkey; 2 Kocaeli University Faculty of Medicine, Department of Pediatric Endocrinology and Diabetes, Kocaeli, Turkey; 3 Kocaeli University Faculty of Medicine, Department of Neonatology, Kocaeli, Turkey

**Keywords:** Metabolic Bone Disease, prematurity, phosphorus, Alkaline phosphatase, Vitamin D

## Abstract

Osteopenia of prematurity has become a common problem recently because of improved survival rates of infants with very low birth weight (VLBW). The incidence of neonatal osteopenia is inversely correlated with gestational age and birth weight. Herein, we present four cases of preterm osteopenia that were referred to the pediatric endocrinology outpatient clinic with diverse clinical and laboratory findings and we discuss the clinical course of these infants with regard to bone disease after discharge from the neonatal intensive care unit (NICU). This report highlights the importance of enteral calcium, phosphorus and vitamin D support at adequate doses following discharge from NICU for preterm infants with VLBW who are at risk of metabolic bone disease.

## INTRODUCTION

Metabolic bone disease of prematurity (MBDP), also called osteopenia of prematurity, has become a common problem recently because of improved survival rates of infants with very low birth weight (VLBW). MBDP is characterized by a reduction in bone mineral content (osteopenia) with or without rachitic changes and several nutritional and biomechanical factors can lead to its development (1). The incidence of neonatal osteopenia is inversely correlated with gestational age and birth weight and its incidence ranges from 30% to 50% in infants with birth weights below 1000 g (2,3,4,5). Risk factors for neonatal osteopenia also include long-term (>4 weeks) total parenteral nutrition (TPN), bronchopulmonary dysplasia (particularly if diuretic treatment has been given), long-term postnatal steroid therapy, necrotizing enterocolitis (particularly cases who underwent surgical intervention) and intolerance to formula or human milk fortified with high mineral content (2). Herein, we present four cases of preterm infants with osteopenia who were referred to our pediatric endocrinology outpatient clinic with diverse clinical and laboratory findings and we aimed to discuss the clinical course of infants with VLBW with regard to bone disease following discharge from a neonatal intensive care unit (NICU).

## 

**Case 1**

**Figure 1:**
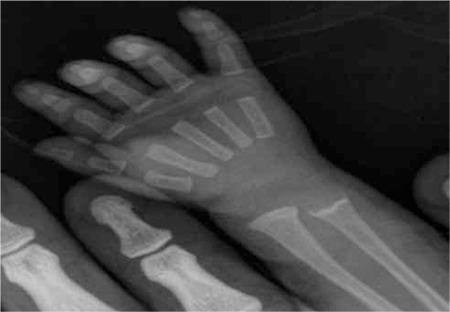
Case 1- wrist X-ray demonstrating metaphyseal cupping and irregularity

This 85-day-old female infant was referred to our clinic because of elevated serum alkaline phosphatase (ALP) levels. She was delivered in the 31st gestational week with a birth weight of 730 g and was admitted to NICU immediately after delivery. The patient underwent surgery for necrotizing enterocolitis on postnatal day 4 and received TPN for 45 days; she was switched to completely enteral feeding eventually on day 46. The infant received mechanical ventilation support for 44 days and was discharged on postnatal day 81. Corrected age was 3 weeks at the time of presentation to the pediatric endocrinology clinic. Her body weight was 2070 g. There was no evidence of rickets on physical examination. She was being fed with preterm formula, supplying 150 mg/kg calcium (Ca) and 95 mg/kg phosphorus (P) daily and was also receiving vitamin D 400 IU D/day. Her serum biochemical parameters were: Ca 9.8 mg/dL, P 4.1 mg/dL, parathyroid hormone (PTH) 617 pg/mL, ALP 1443.5 IU/L, tubular phosphate reabsorption (TRP) rate 98%, 25 hydroxy vitamin D [25(OH)D] 6.1 ng/mL. Maternal 25(OH)D was 17 ng/mL (Table 1). Wrist radiography demonstrated cupping of the metaphyseal margins (Figure 1). Based on these findings, a diagnosis of MBDP was reached. Stoss therapy (150 000

**Figure 2:**
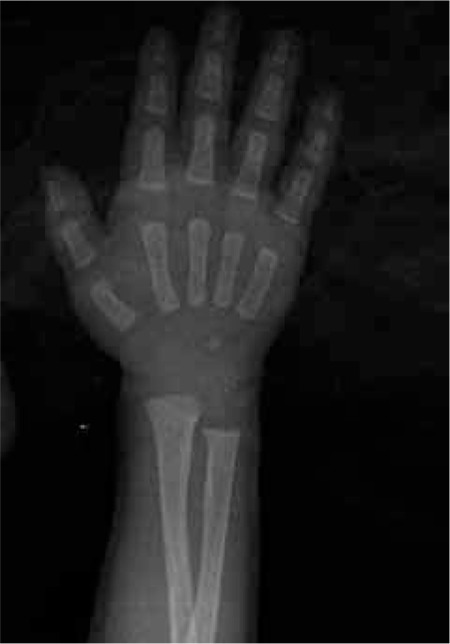
Case 1- improvement of the metaphyseal irregularity on wrist X-ray

IU/ single-dose oral vitamin D3) was administered and a daily dose of 40 mg/kg of supplemental oral phosphate (Sandoz) was initiated. At the end of the first week, laboratory findings were: Ca 9.6 mg/dL, P 6.9 mg/dL, ALP 971 IU/L and PTH 137 pg/mL. The patient’s biochemical and radiological findings (Table 1 and Figure 2) improved at the 8th week of the treatment.

**Case 2**

**Figure 3:**
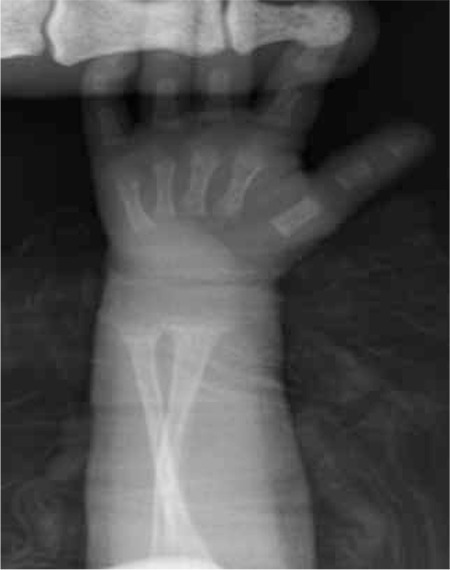
Case 2- wrist X-ray demonstrating metaphyseal widening and irregularity

**Figure 4:**
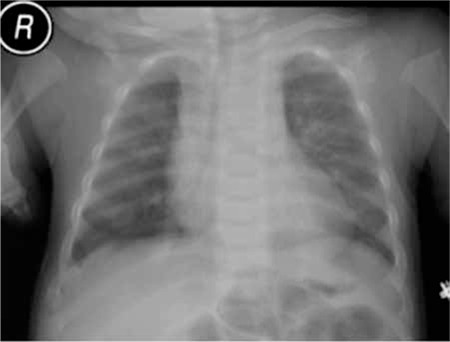
Case 2- chest X-ray demonstrating costo-chondral widening

**Figure 5:**
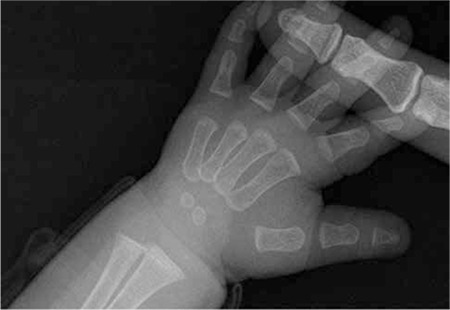
Case 2- improvement of metaphyseal irregularity and widening on wrist X-ray

This 5-month-old female infant was referred to our clinic for hypophosphatemia despite the oral P treatment she was receiving while being treated and monitored for MBDP at the neonatal clinic. She was delivered at a gestational age of 25 weeks and 4 days with a birth weight of 780 g and underwent surgery for necrotizing enterocolitis on postnatal day 15. Because she failed to tolerate oral feedings, she received TPN for 3 months. Her serum P levels began to decrease starting on the 82nd day and the ALP value increased to 1184 IU/L. The patient’s corrected age was 4 weeks and her body weight was 2500 g at the time of referral to Endocrinology clinic. Rachitic rosaries were noted at physical examination. While under treatment with 60 mg/kg/day P, 50 mg/kg/day Ca and 600 U/day vitamin D3, her serum values were: Ca 9.2 mg/dL, P 3.2 mg/dL, ALP 1085 U/L, PTH 147.1 pg/mL and 25(OH)D 43.7 ng/mL (Table 1). Her wrist radiography demonstrated cupping (Figure 3) and thoracic radiography demonstrated costochondral widening (Figure 4). TRP was 98.7%, 1.25-(OH)D was 253 pg/mL. Ca dose was increased to 75 mg/kg/day, P to 90 mg/kg/day and vitamin D to 1000 U/day. Laboratory findings at the end of the first week of treatment were: P 6.7 mg/dL, PTH 61 pg/mL and ALP 1039 U/L. The biochemical and radiological findings improved at the 12th week of treatment (Table 1 and Figure 5).

**Case 3**

This 3-month-old male infant was referred to our clinic for elevated serum ALP level. He was delivered at gestational week 30 with a body weight of 1230 g. He was treated at the NICU for 15 days. At the time of presentation, his corrected age was 3 weeks and body weight was 4070 g. He was receiving breast milk only and 400 IU vitamin D daily. Physical examination did not demonstrate rickets. Serum levels were as follows: Ca 10.5

mg/dL, P 2.5 mg/dL, ALP 1567 IU/L, 25(OH)D 46.1 ng/mL and TRP was 100% (Table 1). P at 60 mg/kg/day was started.

At the end of the first week, serum P levels increased to 7.4

mg/dL, while ALP level dropped to 1396 IU/L. At the 8th week of treatment, biochemical findings improved (Table 1).

**Case 4**

This 84-day-old male infant was also referred for elevated serum ALP level. He was delivered at gestational week 30 with a birth weight of 660 g. He was treated in NICU for 76 days. The patient was switched to enteral feeding at week 2. During his stay in the NICU, he was given an intravenous P solution for his hypophosphatemia. At the time of presentation, his corrected age was 2 weeks and body weight was 1950 g, with no evidence of rickets on physical examination. Serum levels were as follows: Serum P 3 mg/dL, Ca 11 mg/dL, ALP 2035 IU/L, PTH 25.9 pg/mL, 25(OH)D 23.3 ng/mL and TRP 99% (Table 1). There were no relevant radiological findings. The patient was being fed with breast milk only. Oral phosphate treatment with 60 mg/kg/day was initiated and the vitamin D3 dose was increased to 800 IU. His biochemical findings improved at the 45th week of the treatment (Table 1).

## DISCUSSION

Time of appearance of MBDP usually varies between postnatal 6th and 12th weeks. Clinical findings are quite variable. While no symptoms are observed in some cases, others may present with severe bone disease leading even to fractures (6,7). All cases presented herein were asymptomatic and all four patients were referred to our clinic for abnormal laboratory findings such as low serum P and high ALP levels noted at the follow-up visits after discharge from the NICU.

There is an active Ca and P transport from the mother to the fetus throughout pregnancy, which is maximal during the third trimester, particularly between weeks 32 and 36. During this period, Ca transport reaches up to 100-130 mg/kg and P transport up to 60-70 mg/kg daily. Preterm infants are therefore born with inadequate mineral depots. In addition, these infants are also at risk for vitamin D deficiency since there is not enough time for transplacental transfer of vitamin D (2,8,9,10). Given these risk factors with adverse effects on bone health, the recommended daily intake values for enteral feeding for infants with VLBW are 100-220 mg/kg of Ca, 60-140 mg/kg of P and 150-1000 IU of vitamin D (2). Fortified human milk and formulas produced for term infants are not sufficient to supply the mineral needs of preterm infants. Two of the patients (cases 3 and 4) presented here were being fed with only breast milk following discharge from NICU. In patient 2, hypophosphatemia did not resolve despite supplemental Ca and P administration; however, the administered Ca dose was below the recommended level. Oral P dose was close to the lower limit of the recommended dose, failing to elevate the serum P to normal levels. Patient 1 had elevated ALP and reduced P levels despite being fed with preterm formula and having received an appropriate Ca and P intake at recommended doses.

Biochemical parameters (elevated ALP levels, hypophosphatemia) and radiological findings support the diagnosis, although they are not standard approaches for the diagnosis of MBDP (11). An ALP value above 1000 IU/L or higher than 5 times the upper limit of the normal range for adults is an important risk indicator for rickets (7). Interestingly, patient 4 did not have any radiologic findings despite a very high ALP level (2085 IU/L), suggesting that ALP levels and radiological findings do not always correlate.

Serum P levels below 2 mmol/L (6.1 mg/dL) constitute an important risk factor for osteopenia and levels below <1.8 mmol/L (5.4 mg/dL) were found to be related with positive radiological findings (7). The combination of hypophosphatemia and elevated ALP is a highly sensitive indicator in identifying infants at risk of MBD.

Low serum Ca levels do not have a diagnostic or screening value for MBDP. Ca levels may even be higher than normal due to hypophosphatemia (7). As a matter of fact, the fourth case presented here had serum Ca levels higher than normal, which returned to normal following P replacement.

Serum 25(OH)D level is not one of the parameters recommended when considering a diagnosis of MBDP (5,7). Although it has been suggested that Ca absorption was independent from vitamin D during the first month in preterm infants, this has not been confirmed to date (2). There are a very few studies investigating vitamin D status in preterm infants. Two separate studies with infants of mothers of Arabic origin in India (12) and in USA (13) showed increased frequency of vitamin D deficiency in infants with low birth weight. To date, there have been no studies investigating vitamin D status in preterm infants in Turkey, despite reports showing that maternal and neonatal vitamin D deficiency is common in this country (14,15,16,17). Additionally, since these infants are born before vitamin D transfer from the mother to the fetus has been completed; it is highly probable that vitamin D deficiency will be common among them. Considering all these risk factors, we recommend that serum 25(OH)D should be measured, especially in cases whose biochemical parameters are not improving (persisting ALP elevation and hypophosphatemia) despite appropriate Ca and P supplementation. The first case presented here who also had severe vitamin D deficiency, had persisting hypophosphatemia and radiologically confirmed rickets despite treatment with Ca and P at recommended doses. Her serum ALP and PTH levels decreased and P levels reached the desired levels after administration of stoss vitamin D therapy.

Routine measurement of serum PTH or of 25(OH)D levels is not recommended for diagnosis or screening of MBDP (5,7). In a recently published study, secondary hyperparathyroidism was reported in three fourths of infants with VLBW who had radiological evidence of osteopenia and it was suggested that PTH could be a marker of bone demineralization in the newborn (18). It should be kept in mind that secondary hyperparathyroidism may also be seen due to the high incidence of maternal and neonatal vitamin D deficiency in countries like Turkey (14,15,16,17). However, the reference range to be used for PTH levels for preterm infants is unknown (18) and this creates difficulties in interpretation. The very high PTH level observed in our first case (617 pg/mL) proved to be associated with severe vitamin D deficiency [25(OH)D 6.1ng/mL] and showed a rapid decrease after vitamin D stoss therapy. On the other hand, serum 25(OH)D level was normal in the second case who had a high serum PTH level. On speculation, a negative Ca balance secondary to inadequate intestinal absorption of Ca was also considered as a possible cause of high PTH level in case 2. 

In conclusion, enteral Ca and P support at adequate doses following discharge from NICU should be ensured for preterm infants with LBW who are at risk of MBD. Risk of vitamin D deficiency among preterm infants should not be ignored. These infants should be monitored closely not only by neonatologists but also by pediatric endocrinologists.

## Figures and Tables

**Table 1:**
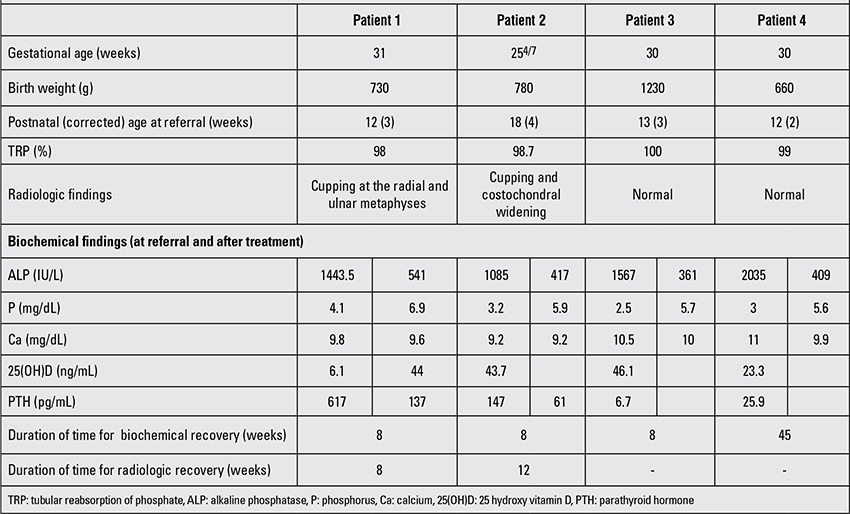
Clinical and biochemical features of the patients
